# Blood-Based Whole-Genome Methylation Analysis of Yili Horses Pre- and Post-Racing

**DOI:** 10.3390/ani15030326

**Published:** 2025-01-24

**Authors:** Jianwen Wang, Wanlu Ren, Zexu Li, Shikun Ma, Luling Li, Ran Wang, Yaqi Zeng, Jun Meng, Xinkui Yao

**Affiliations:** 1 College of Animal Science, Xinjiang Agricultural University, Urumqi 830052, China; wjw1262022@126.com (J.W.); 13201295117@163.com (W.R.); 13593312012@163.com (Z.L.); 18299152719@163.com (S.M.); 18996888638@163.com (L.L.); 17590811761@163.com (R.W.); xjauzengyaqi@163.com (Y.Z.); 2 Xinjiang Key Laboratory of Equine Breeding and Exercise Physiology, Urumqi 830052, China

**Keywords:** whole-genome sequencing, DNA methylation, differentially methylated genes, racing performance, Yili horse

## Abstract

This study aims to analyze the whole-genome DNA methylation differences in Yili horses before and after racing, with the goal of identifying differentially methylated genes associated with racing performance and exploring the epigenetic mechanisms underlying exercise in horses. Blood samples were collected from the jugular veins of the top 3 Yili horses in a 5000 m race, which included 25 competitors, both prior to and within 5 min after the race. Genomic DNA was extracted, followed by sequencing using Whole-Genome Bisulfite Sequencing (WGBS) to assess DNA methylation levels, differentially methylated regions (DMRs), and differentially methylated genes (DMGs). Gene Ontology (GO) and Kyoto Encyclopedia of Genes and Genomes (KEGG) pathway enrichment analyses were performed on the identified DMGs to select candidate genes potentially associated with equine exercise. A total of 18,374 differentially methylated CG regions, 254 differentially methylated CHG regions, and 584 differentially methylated CHH regions were identified. A total of 4293 DMGs were anchored in gene bodies and 2187 DMGs in promoter regions. Functional analysis revealed that these DMGs were mainly enriched in terms related to binding and kinase activity, as well as pathways such as PI3K-Akt signaling and Kaposi sarcoma-associated herpesvirus infection. Further analysis indicated that genes such as *IFNAR2*, *FGF4*, and *DGKH* could be potential candidate genes associated with equine athletic performance. The findings of this study contribute to understanding the epigenetic regulatory mechanisms of equine athletic performance, providing a reference for further in-depth research on horse racing.

## Simple Summary

With the rapid development of horse racing, there is growing interest in related fields such as genetic breeding, training, and animal nutrition. In this study, jugular venous blood samples were collected from the top three Yili horses participating in a 5000 m speed race, both before and immediately after the event, for DNA methylation. The identified methylation regions were primarily enriched in terms related to binding and kinase activity. Pathways such as PI3K-Akt signaling and Kaposi’s sarcoma-associated herpes virus infection, as well as genes like *IFNAR2*, *FGF4*, and *DGKH*, may serve as potential candidates associated with the racing performance in Yili horses. Our findings provide new insights into the epigenetic mechanisms underlying horse racing and offer valuable reference data for identifying new candidate genes related to performance in horse racing.

## 1. Introduction

The Yili is one of the primary racehorse breeds in China, developed through the crossbreeding of Kazakh horses with Thoroughbreds, Arabian horses, and Akhal-Teke, among others. The Yili horses used in racing were primarily derived from the cross between Kazakh horses and Thoroughbreds. Racing is one of the primary uses of horses, generating significant economic value. Understanding the physiological responses of horses during races is essential to ensure their health and welfare. Previous studies have reported the hematological and physiological changes in horses participating in different types of competitions, including endurance races [1,2], three-barrel races [3], racing [4], and trotting [5]. However, the epigenetic mechanisms involved in the racing process of athletic horses remain unclear.

There is a strong correlation between exercise and epigenetic modifications. Exercise-induced epigenetic modifications can influence gene expression and metabolic levels, with the effects varying depending on the type, intensity, and duration of the exercise. Studies have shown that exercise triggers the release of numerous extracellular molecules from various glands, tissues, and organs. These molecules activate intracellular activity groups through multiple mechanisms, including changes in intracellular protein activity, transcription factor binding or displacement, and translocation of key molecules. Such mechanisms lead to changes in the expression of key genes that enable horses to adapt to exercise stress [6]. For instance, genes related to transcription factor activity, oxidoreductase activity, and protein binding showed significant changes in skeletal muscle before and 4 h after exercise in thoroughbred horses [7]. Genes regulated by acute exercise or long-term training are mainly enriched in pathways such as the TCA cycle, oxidative phosphorylation, and pyruvate metabolism [8]. Single aerobic exercise sessions are significantly associated with demethylation of key metabolic genes (e.g., *TFAM*, *PDK4*) involved in metabolism and mitochondrial biosynthesis in skeletal muscle [9]. Blood analysis before and 2 h after a 90 km race in Arabian horses also revealed significant changes in gene expression related to cell migration, energy metabolism, and muscle remodeling [10]. Moreover, whole-blood epigenetic modifications induced by exercise training have been shown to be associated with improvements in human cardiopulmonary function and running performance [11]. Research on racing horses has also revealed significant methylation changes in pathways related to the actin cytoskeleton and other exercise-related pathways such as PI3K-Akt [12].

In summary, previous studies suggest a close relationship between exercise and epigenetic modifications in horses. However, the mechanisms by which exercise-induced DNA methylation changes impact athletic adaptation in horses remain unclear. This study collected blood samples from Yili horses before and immediately after a 5000 m race and analyzed the DNA methylation profiles of these samples using WGBS. By identifying methylation regions or genes potentially associated with exercise in horses, this research provides a reference for understanding the epigenetic mechanisms related to athletic performance in horses.

## 2. Materials and Methods

### 2.1. Ethics Statement

This study was conducted in accordance with the Declaration of Helsinki and approved by the Ethics Committee of Xinjiang Agricultural University (protocol code: 2024003).

### 2.2. Sample Collection

A 5000 m speed race for Yili horses was organized. All participating horses were required to arrive at the race venue by 18:00 the day before the race. Jugular vein blood samples from all participating horses were collected using EDTA venous vacuum tubes by 20:00 the day before the race. Before the race, the breed of each horse was confirmed with their passport information, and only horses that passed a veterinary health inspection were allowed to participate. A total of 24 Yili horses, aged 4 to 5 years, participated in the racing. All horses underwent training similar to that of Thoroughbreds before the racing. Jugular vein blood samples were collected within 5 min post-race from the top three finishers, all of which were 4-year-old stallions. Pre-race blood samples from these top 3 finishers were also used for this study. All samples were immediately stored in liquid nitrogen after collection for future use.

### 2.3. Library Preparation

Genomic DNA was extracted from the blood samples using a DNA extraction kit (Sangon Biotech, Shanghai, China). The concentration and quality of the DNA were assessed using a NanoDrop spectrophotometer (NanoDrop Technologies, Wilmington, DE, USA) and gel electrophoresis. After the sample passed quality control, 100 ng of genomic DNA was mixed with 0.5 ng of unmethylated lambda DNA. The DNA was then sonicated into 200–400 bp segments using a Covaris S220 sonicator (Covaris, Woburn, MA, USA). The unmethylated cytosines were converted to uracil using the EZ DNA Methylation-Gold™ Kit (Zymo Research, Irvine, CA, USA). Subsequently, adapter ligation, fragment selection, and PCR amplification were performed to construct the library. The quality of the library was assessed using the Agilent 5400 system (Agilent, Santa Clara, CA, USA). The qualified library was sent to Novogene (Beijing, China) for paired-end sequencing using the Illumina platform (Illumina, CA, USA).

### 2.4. Bioinformatics Analysis

The quality of the raw data obtained from the Illumina platform was evaluated using FastQC (fastqc_v0.11.8). The software fastp (fastp 0.23.1) was used to filter out adapter contamination and low-quality data from the raw data, generating clean reads. The clean reads were aligned to the horse reference genome (Equus_caballus.EquCab3.0.) using Bismark software (version 0.24.0). The aligned sequences were considered target sequences for subsequent standard and customized analyses, and whole-genome methylation information was extracted from the target sequences.

The data filtering criteria were as follows: remove reads contaminated by adapters. Remove reads with a base quality below 3 at either end or reads containing “N” (indicating undetermined bases). Remove low-quality reads using a sliding window approach, with a window size of 4 bases. If the average base quality in a window was below 15, the read was trimmed from that position onward. Discard reads shorter than 36 nt after trimming; discard reads that could not form paired reads. For single-cell methylation and low-input methylation libraries, the first 10 bp were trimmed from the start of the reads.

### 2.5. Calculation of Cytosine Methylation Levels

Following Krueger’s method [13], Bismarck was used for methylation site detection (https://github.com/FelixKrueger/Bismark, accessed on 21 March 2024). To identify methylation sites, a binomial test was performed using the number of methylated cytosines (mC), the total number of cytosine sites (mC + umC), and the non-conversion rate (r). Sites were considered methylated when the *p*-value, adjusted by false discovery rate (FDR) correction, was less than 0.05. The methylation level of a single site was calculated using the formula: methylation level at a single site = (Reads (mC)Reads (mC + umC)) × 100 [14]. Methylation sites in different gene elements for each sample were visualized using Bedtools software V2.31.1.

### 2.6. Differential Methylation Analysis

Differentially methylated regions (DMRs) were analyzed using DDS software (version 2.12.0). The selection criteria for DMRs were as follows: the proportion of differential methylation sites with a *p*-value smaller than 10^−5^ exceeded 50% within a region; the number of sites in the region was greater than 3; and the length of the region was greater than 50 bp. Two DMRs were merged if the distance between them was less than 100 bp. DMRs were then anchored to genes based on the following standards: for gene regions, a DMR overlapping a gene region (from TSS to TES) by more than 1 bp was considered an anchored region, and the corresponding gene was identified as the anchored gene. For gene promoters, a DMR overlapping the 2 kb region upstream of a gene’s TSS by more than 1 bp was considered an anchored region, and the corresponding gene was identified as the anchored gene [15,16].

### 2.7. Enrichment Analysis of DMGs

Genes anchored to the DMRs were selected for GO and KEGG pathway enrichment analysis. GO enrichment analysis was performed using the GOseq package in R, with a *p*-value < 0.05 considered significant. KEGG pathway enrichment analysis was performed using KOBAS (version 3.0) software, with a *p*-value < 0.01 considered significant.

## 3. Results

### 3.1. Overview of DNA Methylation

The statistical results of whole-genome bisulfite sequencing for three blood samples from Yili horses before and after the race are shown in Table 1. In the pre-race group, each sample had an average of 302,079,263 reads, which, after filtering out low-quality data, yielded 298,111,640 clean reads. In the post-race group, each sample had an average of 344,527,333 reads, with 340,023,453 clean reads remaining after filtering. The average clean rate for both groups was 98.69%. Mapping the clean reads to the horse reference genome resulted in 267,310,135 and 303,202,343 mapped reads, with mapping rates of 89.67% and 89.05%, respectively.

The genome’s DNA methylation mainly occurs in three contexts (CG, CHG, and CHH), with the CG context having the highest methylation rate. The CHG and CHH contexts showed very low or nearly zero methylation levels. The post-race group exhibited a higher CG methylation rate than the pre-race group, while the CHG and CHH methylation rates were lower in the post-race group compared to the pre-race group.

**Figure 1:**
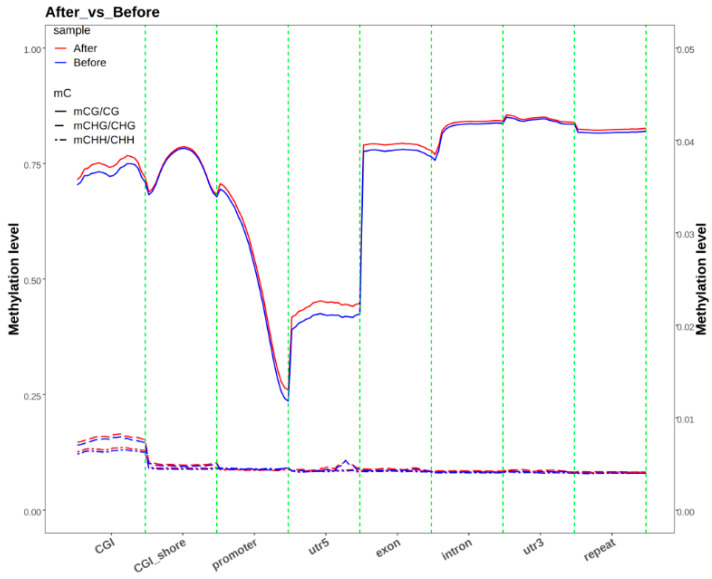
Distribution of methylation levels on gene functional elements.

To further compare methylation levels in different functional genomic elements and gene upstream/downstream regions between the pre-race and post-race groups, we analyzed methylation levels in the promoter, exon, intron, CGI, CGI shore, and repeat regions. The results showed no significant differences in methylation levels within the same functional region or transcriptional elements between the two groups (Figure 1).

**Figure 2:**
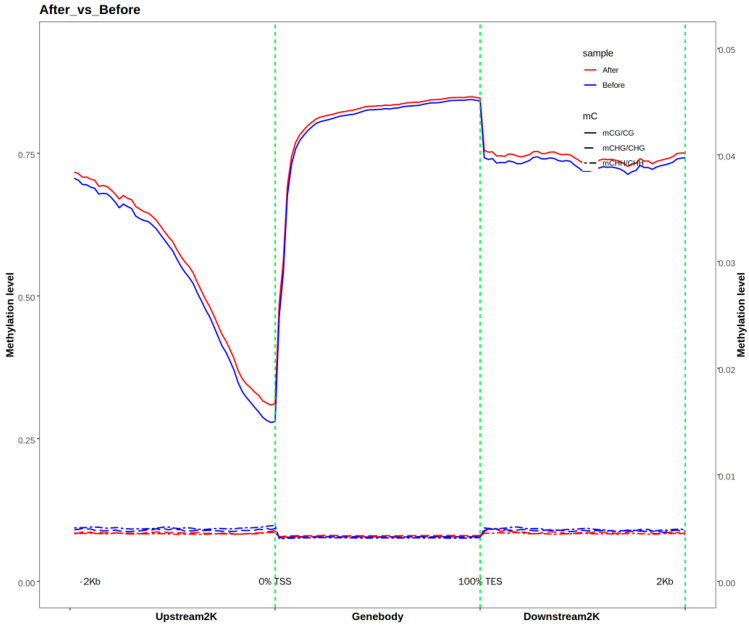
Distribution of methylation levels in the upstream and downstream 2 K regions of genes.

Methylation levels in the CG context were higher than those in the CHG and CHH contexts, where all functional elements exhibited low and stable methylation, except for the relatively higher methylation in the CGI region. In the CG context, the methylation level was lowest in the 5′ UTR region, while the 3′ UTR, repeat, and intron regions had higher methylation levels. Among the transcriptional elements, the intron region had higher methylation levels than the exon region. A comparison of methylation levels in the upstream/downstream 2 K regions of the genome (Figure 2) showed that, in the CG context, methylation levels were lowest at the TSS and highest at the TES, with a gradual increase from TSS to TES. Methylation levels in the CHG and CHH contexts were very low across all regions.

### 3.2. DMR Analysis

**Figure 3:**
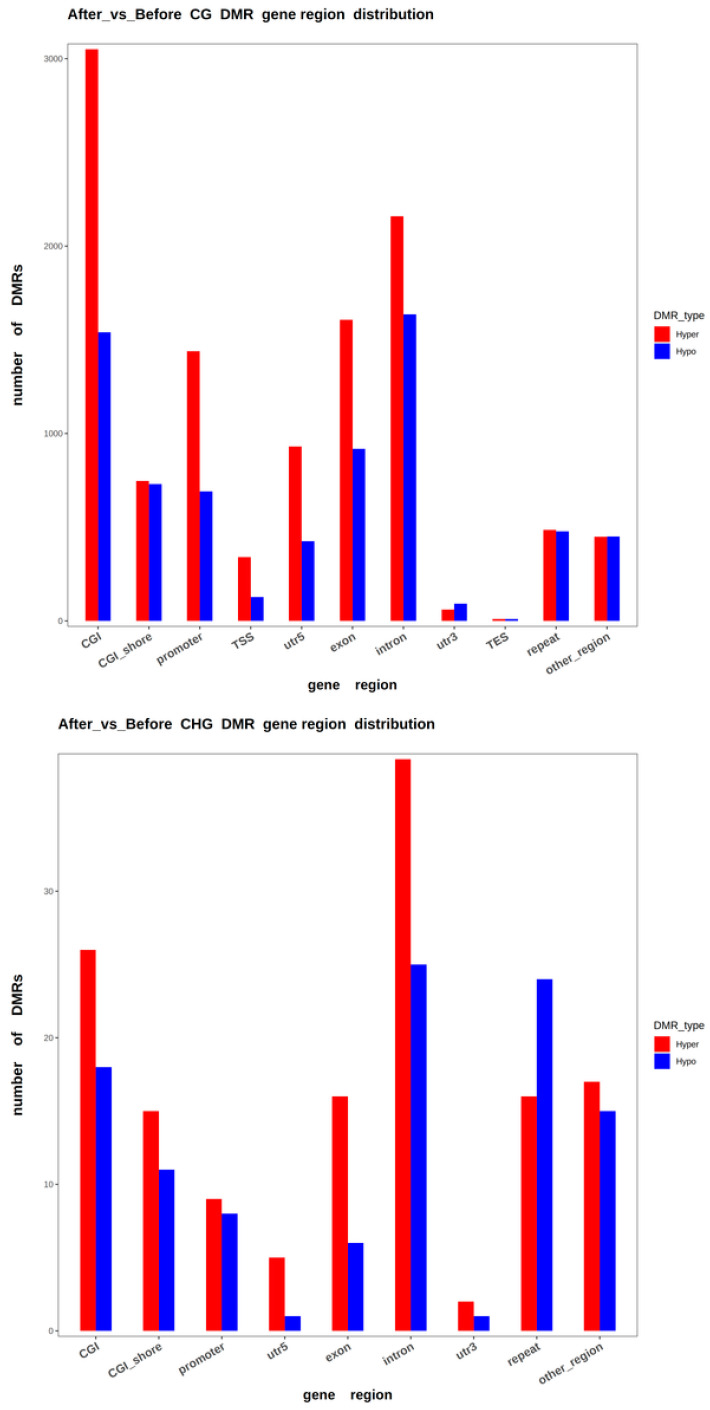

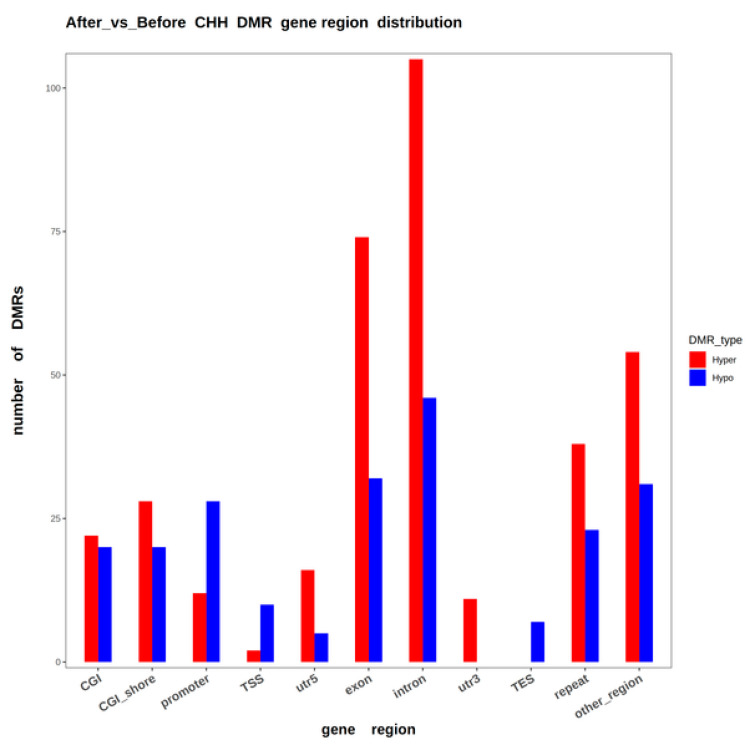
Distribution of DMRs in various functional genomic elements.

A total of 18,374 differentially methylated CG regions, 254 differentially methylated CHG regions, and 584 differentially methylated CHH regions were identified. Among these DMRs, 11,784 regions (CG: 11,277; CHG: 145; CHH: 362) were hypermethylated, while 7428 regions were hypomethylated (CG: 7097; CHG: 109; CHH: 222). The identified DMRs were annotated to equine genomic elements such as promoter, exon, intron, CGI, CGI shore, repeat, TSS, and TES. The majority of DMRs were located in CGI regions, followed by introns, exons, promoters, CGI shores, 5′ UTRs, and repeat regions, with relatively fewer DMRs in other areas (see Figure 3 and Table 2).

**Figure 4:**
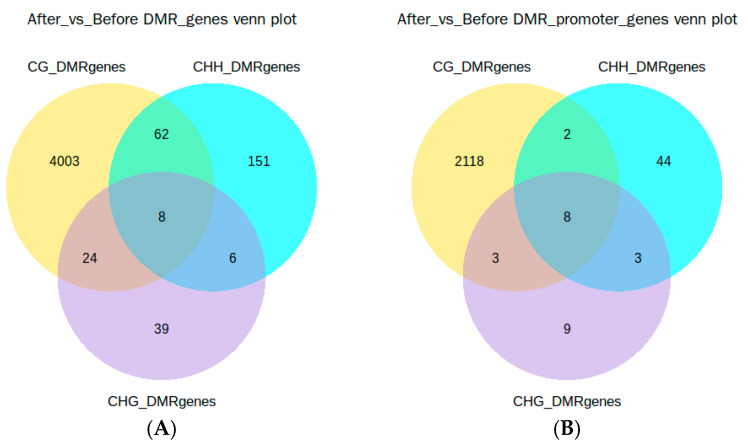
Venn diagrams of genes anchored to DMRs. (**A**) Anchored Genes; (**B**) genes related to anchored promoter regions.

The identified DMRs were anchored to genes (from TSS to TES) and promoter regions. The results showed that 4293 genes were anchored (Figure 4A and Table S1), of which 4003 genes were exclusively from the CG region, 39 genes from the CHG region, and 151 genes from the CHH region. Additionally, 24 genes were shared between CG and CHG regions, 62 genes were shared between CG and CHH regions, 6 genes were shared between CHH and CHG regions, and 8 genes were present in all three regions. Furthermore, 2187 genes were anchored to the promoter regions (Figure 4B and Table S1), of which 2118 genes were exclusively from the CG region, 9 from the CHG region, and 44 from the CHH region. There were three genes shared between the CG and CHG regions, two shared between the CG and CHH regions, three shared between the CHG and CHH regions, and eight genes were shared across all three regions.

### 3.3. Functional Enrichment Analysis of DMGs

**Figure 5:**
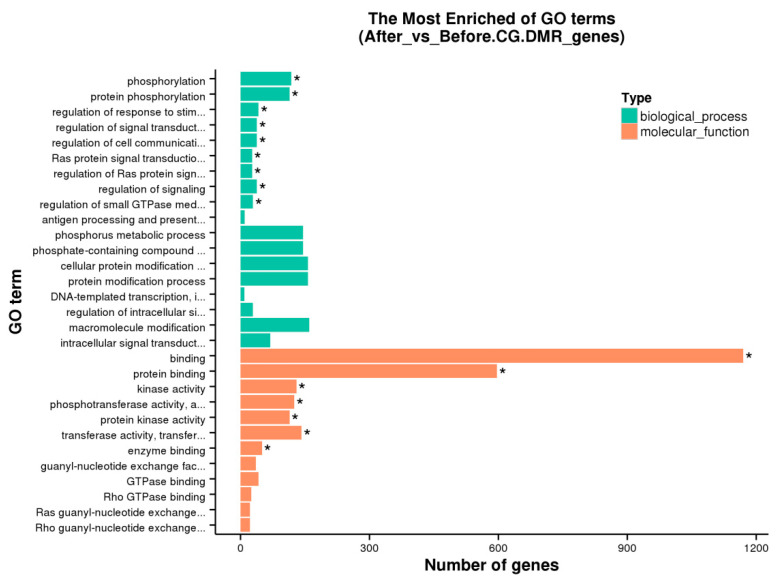

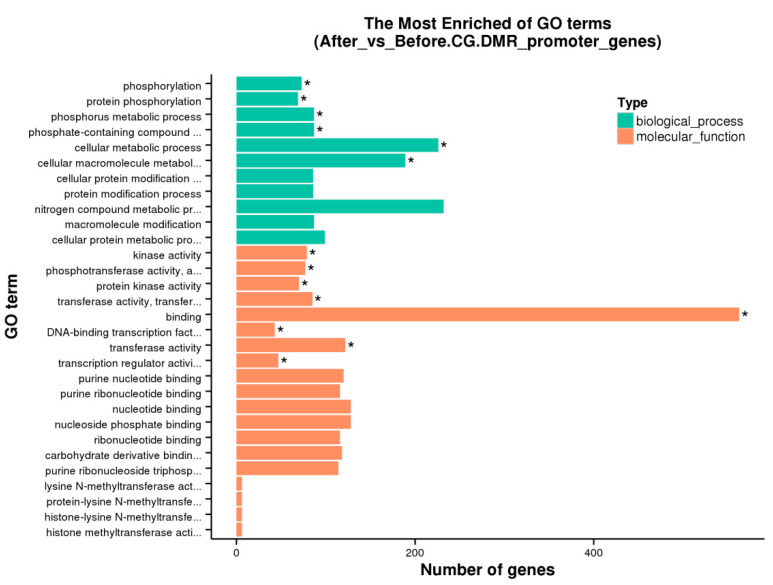
Bar charts of enriched GO terms. * Corrected *p*-value < 0.05.

GO and KEGG enrichment analyses were performed on the anchored genes. Since most DMRs belong to the CG context, enrichment analysis was conducted only for CG methylation. Based on the GO database, 16 GO terms were significantly enriched in the gene body region (from TSS to TES) (corrected *p*-value < 0.05), while 14 GO terms were significantly enriched in the promoter region (corrected *p*-value < 0.05). The main GO terms enriched in both the gene body and promoter regions included binding, kinase activity, phosphorylation, protein phosphorylation, phosphotransferase activity (alcohol group as acceptor), protein kinase activity, and transferase activity (transferring phosphorus-containing groups). The top 30 GO terms, ranked by corrected *p*-value in ascending order, were selected for display (Figure 5 and Table S2).

**Figure 6:**
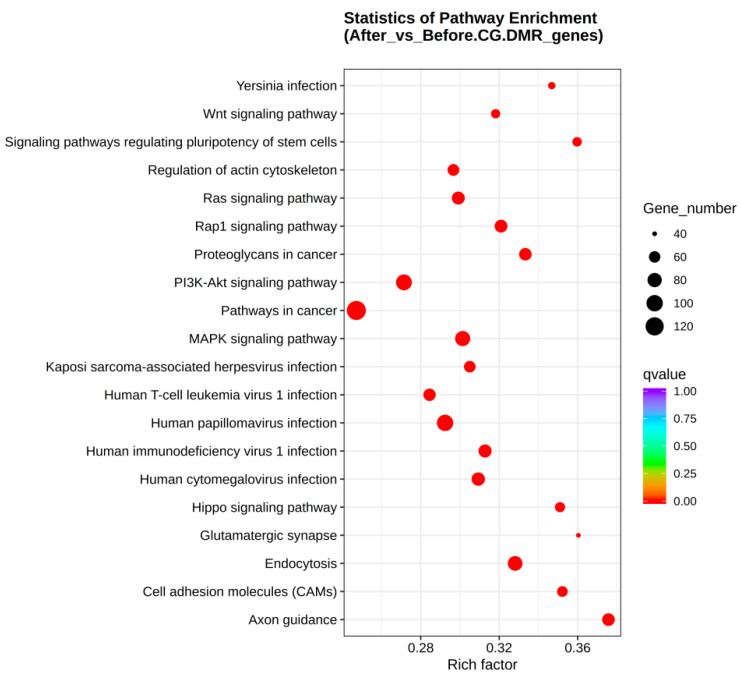
Scatter plot of enriched KEGG pathways.

For the KEGG enrichment analysis, 98 pathways were significantly enriched in the gene body region (from TSS to TES) (corrected *p*-value < 0.01), and 107 pathways were significantly enriched in the promoter region (corrected *p*-value < 0.01). The top 20 most significantly enriched pathways were selected for display (Figure 6 and Table S2). The pathways enriched in both the gene body and promoter regions included human papillomavirus infection, PI3K-Akt signaling pathway, and Kaposi sarcoma-associated herpesvirus infection.

Combining the localization of whole-genome DMRs with functional analysis of DMGs, it was found that most of the genes enriched in GO and KEGG analyses were located in intron regions. Further screening was conducted on the genes significantly enriched in GO and KEGG, revealing that *IFNAR2*, *FGF4*, and *DGKH* may be related to equine athletic performance.

## 4. Discussion

In this study, we used WGBS to analyze the DNA methylation profiles of Yili horses before and immediately after a race to explore the effects of racing on DNA methylation, aiming to identify methylation-related genes associated with racing. Our results showed that the majority of methylation occurred in the CG context, while methylation levels in the CHH and CHG contexts were relatively low. Previous studies have confirmed that the DNA methylation pattern in mammals is predominantly CG based [17], and methylation levels in the CHH and CHG contexts are relatively low, indicating a certain degree of conservation in DNA methylation patterns. The findings from the blood methylation study conducted on Thoroughbreds, both before and after exercise, indicated that CG methylation significantly increased in superior males following exercise, whereas a decrease in methylation was observed in inferior females post-exercise [18]. A comparison of the methylation patterns across different tissues between thoroughbred and Jeju horses demonstrated that the CG-methylation levels in thoroughbred horses were higher than those in Jeju horses [19]. Given that Thoroughbreds predominantly compete in speed races and Jeju horses excel in endurance events, it is plausible that participation in speed races may contribute to elevated CG methylation levels in horses. Consistent with prior research, this study found an increase in CG-type methylation in Yili horses after racing. These results support the notion that exercise induces changes in methylation patterns in horses. Additionally, we found higher methylation levels in the 3′-UTR, repeat, and intron regions, which is consistent with findings in other animals [20,21,22,23]. The 3′-UTR region contains numerous regulatory elements that play crucial roles in mRNA stability, localization, translation, and mediation of protein interactions, contributing to cellular homeostasis [24,25]. The significant physiological changes that occur in horses shortly after intense exercise [21,26] may explain the higher methylation levels in this region. The repeat region is a key component of gene regulatory networks and plays an important role in gene expression and transcriptional regulation. It is also a potential factor contributing to genomic instability, as structural changes in repeats are often associated with genomic instability [27]. Thus, methylation may serve to maintain chromatin stability in this region [20]. The high proportion of intron regions might also account for the elevated methylation levels observed in introns. Studies have shown relatively low methylation levels in the 5′ UTR, promoter, and TTS regions, which is consistent with our findings [18,19,28].

Existing research indicates that physical exercise can induce epigenetic changes in genes [9]. In this study, we identified the significantly differentially methylated gene *IFNAR2*, which encodes a protein involved in regulating the homeostasis and function of immune cells [29]. This gene plays a crucial role in initiating immune response signal transduction and may protect the body by regulating both innate and adaptive immune responses [30]. Intense exercise can trigger the activation of leukocytes, leading to the release of large amounts of pro-inflammatory cytokines and free radicals [31]. Moreover, strenuous endurance exercise can disrupt immune system homeostasis [32], leading to neutrophil-induced leukocytosis and immunosuppression in the systemic circulation [33].

Similar results have been observed in studies on horses [34,35]. This may explain the methylation of the *IFNAR2* gene observed in this study. The gene’s expression is regulated through methylation in both the gene body and promoter regions, modulating immune responses and maintaining cellular homeostasis and function. Further GO enrichment analysis showed that this gene is significantly enriched in the binding term, likely due to the requirement for *IFNAR2* to bind with *JAK2*, which activates related kinases to regulate immune responses [36]. However, this study did not examine the levels of white blood cells, particularly neutrophils, highlighting the need for further research to determine whether their regulation through exercise is associated with immune regulation in Yili horses. KEGG pathway analysis indicated that *IFNAR2* is significantly enriched in the Kaposi sarcoma-associated herpesvirus infection pathway. The latent transcripts in this pathway can drive cell proliferation and prevent apoptosis, enhancing the host cell’s antioxidant defense and anti-inflammatory abilities [37]. The results of functional enrichment analyses suggest that the *IFNAR2* gene may regulate exercise-induced inflammatory responses through the binding term and the Kaposi sarcoma-associated herpesvirus infection pathway, thereby maintaining cellular homeostasis and normal physiological function.

*FGF4* functions in wound healing and tissue regeneration by promoting cell proliferation, migration, and tissue remodeling through the activation of FGF receptors on the cell surface, which initiates a series of downstream signaling pathways in adult tissues [38]. During tissue damage or inflammatory responses, *FGF4* expression increases significantly, accelerating tissue repair and maintaining cellular homeostasis [39]. In this study, the significantly methylated genes identified include the *FGF4* gene, suggesting its potential involvement in tissue regeneration and repair tissue regeneration and repair. Intense acute exercise may cause tissue damage [40,41], and the methylation of the *FGF4* gene may regulate its expression to promote tissue repair and maintain structural stability. Additionally, *FGF4* interacts with other growth factors to promote angiogenesis [42], which is critical for oxygen and nutrient supply during the regeneration process [43]. During a race, horses require substantial energy, necessitating sufficient oxygen and nutrient supply [44,45]. The expression of *FGF4* provides favorable conditions for this process, which could explain why its methylation plays a regulatory role in exercise. In this study, *FGF4* was significantly enriched in the binding term and the PI3K-Akt signaling pathway. This is likely because FGF4 binds to its homologous receptor, a serine/threonine protein kinase, and enters the signaling pathway through multiple intermediate components, such as adaptor proteins and protein tyrosine phosphatases, thereby regulating signal transduction [46]. Furthermore, *FGF4* activates the PI3K-Akt signaling pathway by binding to *FGFR*, regulating cell growth, migration, immune modulation, and angiogenesis [47], thereby maintaining cellular homeostasis, immune function, and energy supply during the race.

The *DGKH* gene is involved in lipid metabolism. Its expression directly affects how efficiently cells utilize lipids, impacting the body’s energy production and storage, which is crucial for maintaining energy balance [48,49]. *DGKH* is also closely linked to glucose metabolism. Studies have shown that it plays a regulatory role in converting glucose into energy, and dysfunction in this gene may lead to glucose metabolism disorders [50]. In this study, significant changes in the methylation levels of the *DGKH* gene were observed in pre-race and post-race samples, likely reflecting the high energy demands during the race. Methylation of this gene regulates its expression to ensure better energy provision for the cells. GO enrichment analysis revealed that *DGKH* was significantly enriched in the kinase activity term. Previous studies have shown that *DGKH* is activated by phosphorylation of the MARCKS domain mediated by protein kinase C (PKC) [51], and its interaction with PKC reduces PKC activity and promotes diacylglycerol metabolism [52]. This could explain the *DGKH*’s enrichment in the kinase activity term, though further research is needed to confirm the regulatory mechanisms.

## 5. Conclusions

This study analyzed the DNA methylation profiles of Yili horses’ blood before and immediately after a 5000 m race. The results showed that differential methylation patterns before and after the race were predominantly CG-type, with a total of 19,212 DMRs identified. These DMRs exhibited higher methylation levels in the 3′ UTR, repeat, and intron regions. Additionally, 4293 and 2187 DMGs were identified in the gene body and promoter regions, respectively. These DMGs were significantly enriched in the binding and kinase activity terms, as well as the PI3K-Akt signaling pathway and the Kaposi sarcoma-associated herpesvirus infection pathway, suggesting that these terms and pathways might be influenced by the racing activity of the horses. Notably, *IFNAR2*, *FGF4*, and *DGKH* were identified as candidate genes potentially associated with the racing performance of Yili horses. It is assumed that the expression of these genes may be suppressed by methylation during racing activities. Our findings provide new insights into the epigenetic mechanisms underlying horse racing and offer reference data for identifying new candidate genes related to horse racing.

## Figures and Tables

**Table 1:** Sequencing information and methylation rates.

Samples	Pre-Race Group	Post-Race Group
Raw reads	302,079,263	344,527,333
Clean reads	298,111,640	340,023,453
Clean rate (%)	98.69	98.69
Mapped reads	267,310,135	303,202,343
Mapped rate (%)	89.67	89.05
mC (%)	4.29	4.41
mCG(%)	70.49	72.53
mCHG (%)	0.083	0.077
mCHH (%)	0.063	0.060

**Table 2:** Distribution of DMRs in CG, CHG, and CHH.

Gene_Region	CG		CHG		CHH	
	Hyper	Hypo	Hyper	Hypo	Hyper	Hypo
CGI	3050	1540	26	18	22	20
CGI_shore	746	730	15	11	28	20
promoter	1439	691	9	8	12	28
TSS	341	127			2	10
utr5	931	425	5	1	16	5
exon	1606	918	16	6	74	32
intron	2159	1636	39	25	105	46
utr3	61	92	2	1	11	0
TES	10	10			0	7
repeat	485	478	16	24	38	23
other_region	449	450	17	15	54	31

## Data Availability

The results of whole-genome methylation sequencing of horse blood have been deposited to the BioProject with the accession number PRJNA1195377. Other data used and analyzed during the current study are available from the corresponding author upon reasonable request.

## Supplementary Materials

The following supporting information can be downloaded at https://www.mdpi.com/article/10.3390/ani15030326/s1, Table S1. Genes Anchored to DMRs-1. Table S2. Genes of enrichment analysis.
